# Hydroxylamine-Enhanced NiFe_2_O_4_/H_2_O_2_ Fenton-like System for Phenol Degradation at an Initial pH of 7: Performance and Mechanistic Insights

**DOI:** 10.3390/molecules31162765

**Published:** 2026-08-09

**Authors:** Hongqiang Yuan, Zheyuan Zhan, Ying Zhang, Shuo Wang, Kang Chen, Xiangyang Huang

**Affiliations:** School of Urban Construction, Yangtze University, Jingzhou 434023, China; hqyuan@yangtzeu.edu.cn (H.Y.); 501153@yangtzeu.edu.cn (Y.Z.); 2022720810@yangtzeu.edu.cn (S.W.); 2023000275@yangtzeu.edu.cn (K.C.); 100991@yangtzeu.edu.cn (X.H.)

**Keywords:** hydroxylamine, H_2_O_2_, NiFe_2_O_4_, phenol degradation, metal-valence transformation

## Abstract

A major limitation hindering the practical application of heterogeneous Fenton-like systems is their inherently slow reaction kinetics, particularly near neutral pH. To address this issue, a hydroxylamine (HA)-enhanced NiFe_2_O_4_/H_2_O_2_ system was developed for phenol degradation. At an initial pH of 7, with 5 mmol/L HA and 10 mmol/L H_2_O_2_, the system achieved 97.8% phenol degradation within 60 min, compared with 17.4% in the HA-free system. XPS analysis showed that the Fe^2+^ proportion increased from 49.1% to 53.6% and the Ni^2+^ proportion increased from 52.1% to 63.4% after reaction. These changes are consistent with HA facilitating the formation or regeneration of reduced metal species, suggesting that HA may be related to accelerated Fe^3+^/Fe^2+^ or Ni^3+^/Ni^2+^ cycling. Radical scavenging experiments and electron paramagnetic resonance (EPR) results indicated that identified hydroxyl (•OH) and superoxide (O_2_•^−^) radicals were the predominant reactive species. Metal-leaching and catalyst-removal experiments indicated that both heterogeneous and homogeneous processes contributed to phenol degradation. The results provide mechanistic insights into HA-enhanced H_2_O_2_ activation while also highlighting the need to consider metal leaching, HA-derived nitrogen products, and catalyst reusability.

## 1. Introduction

Phenol (C_6_H_5_OH), a persistent organic pollutant, is commonly found in industrial wastewater from the petrochemical, pharmaceutical, and pesticide industries. It poses significant environmental and health risks due to its carcinogenicity, bioaccumulation potential, and resistance to natural degradation [1]. Conventional water treatment methods, such as physical adsorption and biodegradation, have shown limited efficiency in phenol removal, particularly at low concentrations (<100 mg/L) or under high-toxicity scenarios [2]. The inherent limitations of these conventional methods have spurred the development of more advanced treatment technologies [3]. As a result, advanced oxidation processes (AOPs) have been extensively investigated as a promising class of technologies for the efficient degradation of refractory organic pollutants [4].

Among various oxidants, hydrogen peroxide (H_2_O_2_) is widely used in AOPs due to its environmentally friendly nature and lack of secondary pollution. In traditional homogeneous Fenton systems, H_2_O_2_ reacts with Fe^2+^ to generate hydroxyl radicals (•OH), which have a high oxidation potential of approximately 2.8 V and can non-selectively oxidize a broad range of organic pollutants [5]. However, homogeneous Fenton systems suffer from several drawbacks, including a narrow effective pH range (typically pH 2–4), substantial iron sludge production, and difficulties in catalyst recovery and reuse [6]. The formation of iron sludge not only deactivates the catalyst but also poses a secondary waste disposal problem, representing a major economic and operational drawback [7]. To overcome these issues, heterogeneous Fenton-like systems employing solid-phase catalysts (e.g., iron oxides, spinels) have been developed for more efficient and sustainable H_2_O_2_ activation [8].

Spinel ferrites (AB_2_O_4_, such as CoFe_2_O_4_ and NiFe_2_O_4_) are attractive Fenton-like catalysts due to their stable crystal structures, tunable electronic properties, and catalytic activity [9]. Their catalytic performance is intrinsically linked to the distribution and redox behavior of cations in the crystal lattice [10]. Among these materials, NiFe_2_O_4_ is particularly promising because of its low cost and high catalytic activity [11]. The activation of H_2_O_2_ in NiFe_2_O_4_-based systems can involve surface valence transformations of Fe and Ni species, accompanied by the generation of reactive oxygen species such as hydroxyl (•OH) and superoxide (O_2_•^−^) radicals [12]. The synergistic interplay between Ni and Fe sites significantly enhances the electron transfer process, leading to more efficient oxidant activation [13]. Nevertheless, under neutral or alkaline conditions, surface Fe^3+^ tends to form inert Fe(OH)_3_, thereby significantly reducing catalytic activity [14]. This deactivation is primarily due to the precipitation of ferric oxyhydroxides on the catalyst surface, which blocks active sites and hinders their interaction with H_2_O_2_ [15].

To enhance the Fe^3+^/Fe^2+^ cycling efficiency, reductants such as hydroxylamine (NH_2_OH, HA) have been introduced. Chen et al. [16] applied HA as a reducing promoter to accelerate Fe^3+^/Fe^2+^ cycling in homogeneous Fenton oxidation, thereby improving benzoic acid degradation by 2.8-fold. Subsequently, Hou et al. [17] extended this strategy to a heterogeneous α-FeOOH/H_2_O_2_ system, achieving efficient degradation of various organic pollutants at pH 5.0.

However, the influence of HA on the surface valence states of Fe and Ni in NiFe_2_O_4_-based Fenton-like systems remains insufficiently understood. In particular, changes in the Ni oxidation state may arise from direct reduction with HA, Fe-mediated electron transfer, or electron redistribution through Fe-O-Ni linkages, and therefore the observed ex situ valence-state changes should be interpreted cautiously [18,19].

In this study, the primary objective was to investigate how HA influences H_2_O_2_ activation and surface metal-valence transformations in the NiFe_2_O_4_/H_2_O_2_ system. Phenol degradation, Fe and Ni valence-state changes, reactive species, and degradation intermediates were examined. Metal leaching, homogeneous and heterogeneous contributions, nitrogen-containing transformation products, and catalyst reusability were evaluated as complementary indicators of practical performance.

This work provides mechanistic insight into HA-enhanced H_2_O_2_ activation by Ni-Fe oxide catalysts and identifies practical limitations that should be considered in further development.

## 2. Materials and Methods

### 2.1. Experimental Materials

The reagents used in this study, including ferric chloride hexahydrate (FeCl_3_·6H_2_O), nickel nitrate tetrahydrate (Ni(NO_3_)_2_·4H_2_O), hydroxylamine hydrochloride (NH_2_OH·HCl), phenol (C_6_H_5_OH), sulfuric acid (H_2_SO_4_), sodium hydroxide (NaOH), methanol, benzoquinone (BQ), tert-butyl alcohol (TBA), were all of analytical grade and purchased from China National Pharmaceutical Group Chemical Reagent Co., Ltd. (Shanghai, China). Deionized water (resistivity ≥ 18.2 MΩ·cm) was used in the experiments.

### 2.2. Preparation of Catalysts

A simple hydrothermal method was employed for the synthesis of NiFe_2_O_4_ nanoparticles, following the procedure reported in our previous study [20]. Typically, 1.08 g of FeCl_3_·6H_2_O and 0.49 g of Ni(NO_3_)_2_·4H_2_O were co-dissolved in 50 mL of deionized water at room temperature. The pH of the solution was adjusted to 12 by adding 3 mol/L NaOH. The precursor solution was then sealed in a Teflon-lined autoclave and subjected to hydrothermal treatment at 180 °C for 12 h, yielding a red-brown precipitate. The obtained solid was washed repeatedly with distilled water and ethanol until the filtrate became neutral. Finally, the product was dried in an oven. The dried material was ground into a fine powder to obtain the NiFe_2_O_4_ catalyst.

### 2.3. Catalyst Characterization

The crystal structure of the catalyst was analyzed by X-ray diffraction (XRD, Bruker D8 Advance, Billerica, MA, USA) with Cu Kα radiation (λ = 0.15406 nm), operated at 40 kV and 40 mA. The catalyst’s morphology was characterized using a ZEISS GeminiSEM 300 scanning electron microscope (SEM, Jena, Germany) operated at 3 kV. Transmission electron microscopy (TEM, FEI Talos F200X, Hillsboro, OR, USA) was employed to examine the morphology and microstructure. X-ray photoelectron spectroscopy (XPS, Thermo Scientific K-Alpha, Waltham, MA, USA) with Al Kα radiation (*hv* = 1486.6 eV) and a 400 μm spot size was used for the analysis of surface chemical composition and oxidation states of the elements. The intermediate products were identified by liquid chromatography-mass spectrometry (LC-MS, Thermo Scientific, Waltham, MA, USA). Particle-size distributions were obtained from multiple representative SEM and TEM images using ImageJ (version 1.54g; National Institutes of Health, Bethesda, MD, USA). Equivalent circular diameters were measured for 200 clearly distinguishable SEM agglomerates and 200 clearly distinguishable TEM primary particles, while objects touching the image boundaries or exhibiting severe overlap were excluded. For the TEM analysis, only clearly distinguishable near-equiaxed particles were included. The SEM and TEM specimens were prepared from aliquots of the same NiFe_2_O_4_ synthesis batch.

### 2.4. Degradation Experiments

The phenol degradation experiments were performed in a multi-channel reaction apparatus to evaluate the degradation efficiency of the HA-enhanced NiFe_2_O_4_/H_2_O_2_ Fenton-like system. All experiments were conducted in the dark without external UV or visible-light irradiation. The experimental procedure was as follows: 50 mL of a phenol solution was placed in a quartz glass reactor, followed by the addition of 0.6 g/L of the NiFe_2_O_4_ catalyst. This concentration was selected to simulate high-strength industrial wastewater streams (typically ranging from 50 to 1000 mg/L in petrochemical and coking effluents) [21], rigorously evaluating the oxidative robustness of the system under high pollutant loading. The reaction temperature was maintained at 22 ± 1 °C. For the time-resolved pH measurements, the initial pH of each reaction system was adjusted to 7.00 before HA and H_2_O_2_ addition. The pH was recorded at 0, 5, 10, 20, 30, 40, and 60 min using a Raycom PHS-3C pH meter (Shanghai INESA Scientific Instrument Co., Ltd., Shanghai, China), without further adjustment during the reaction. Unless otherwise stated, all experiments were independently performed in triplicate, and the results are presented as the mean ± standard deviation in the figures. Prior to initiating the reaction, the mixture was continuously stirred at 500 rpm for 30 min to achieve adsorption equilibrium. Subsequently, specified amounts of HA and H_2_O_2_ were added to initiate the reaction. The H_2_O_2_ concentration of 10 mmol/L corresponds to approximately 340 mg/L. Aliquots of 900 µL were collected at predetermined time intervals (0, 5, 10, 20, 30, 40, and 60 min). The degradation reaction in each sample was immediately quenched by the addition of 100 µL of methanol. Then the samples were filtered through 0.45 μm membrane filters. The phenol concentrations of the samples were determined by HPLC (Shimadzu LC-16) using a C18 reversed-phase column (Inertsil ODS-3, Shimadzu, Kyoto, Japan). The mobile phase was methanol/water (40:60, *v*/*v*) at a flow rate of 1.0 mL min^−1^, and the detection wavelength was set to 270 nm.

### 2.5. Additional Analytical and Control Experiments

For metal leaching measurements, reaction samples were centrifuged and filtered through 0.22 μm membranes before analysis. Dissolved Fe and Ni were determined colorimetrically using a UV–Vis spectrophotometer (Shimadzu Instruments (Suzhou) Co., Ltd., Suzhou, China). Control experiments with Fe^3+^/H_2_O_2_/HA and Fe^3+^/H_2_O_2_ were conducted to evaluate the contribution of dissolved Fe species. In the catalyst-removal experiment, the NiFe_2_O_4_ catalyst was removed after 10 min by centrifugation and filtration, and phenol degradation in the remaining solution was monitored until 60 min.

Residual NH_2_OH-N and nitrogen-containing products (NH_4_^+^-N, NO_2_^−^-N, and NO_3_^−^-N) were measured at 0, 10, 20, 40, and 60 min using a UV–Vis spectrophotometer, with all concentrations expressed as mg N/L.

EPR measurements were performed using a Bruker EMXplus-6/1 EPR spectrometer (Bruker BioSpin GmbH & Co. KG, Ettlingen, Germany), with DMPO as the spin-trapping agent. The DMPO solution was supplied and prepared by the testing facility. For sample preparation, 30 μL of the reaction solution was mixed with 30 μL of 100 mmol/L DMPO solution. Key acquisition parameters included: center field, 3500 G; sweep width, 100 G; microwave frequency, approximately 9.82 GHz; microwave power, 6.325 mW; modulation frequency, 100 kHz; modulation amplitude, 1.0 G; sweep time, 30 s; receiver gain, 30 dB; and temperature, 295 K. DMPO–•OH spectra were recorded at 0 and 10 min, while DMPO–O_2_•^−^ spectra were recorded at 5 and 10 min.

After each reuse cycle, the catalyst was recovered by centrifugation, washed with deionized water, dried at 60 °C to constant mass, and reused without adding fresh catalyst. The single-cycle recovery yield was calculated from the catalyst masses before and after each cycle, and the cumulative mass retention was calculated relative to the initial catalyst mass.

## 3. Results and Discussion

### 3.1. Characterization of the Catalyst

**Figure 1:**
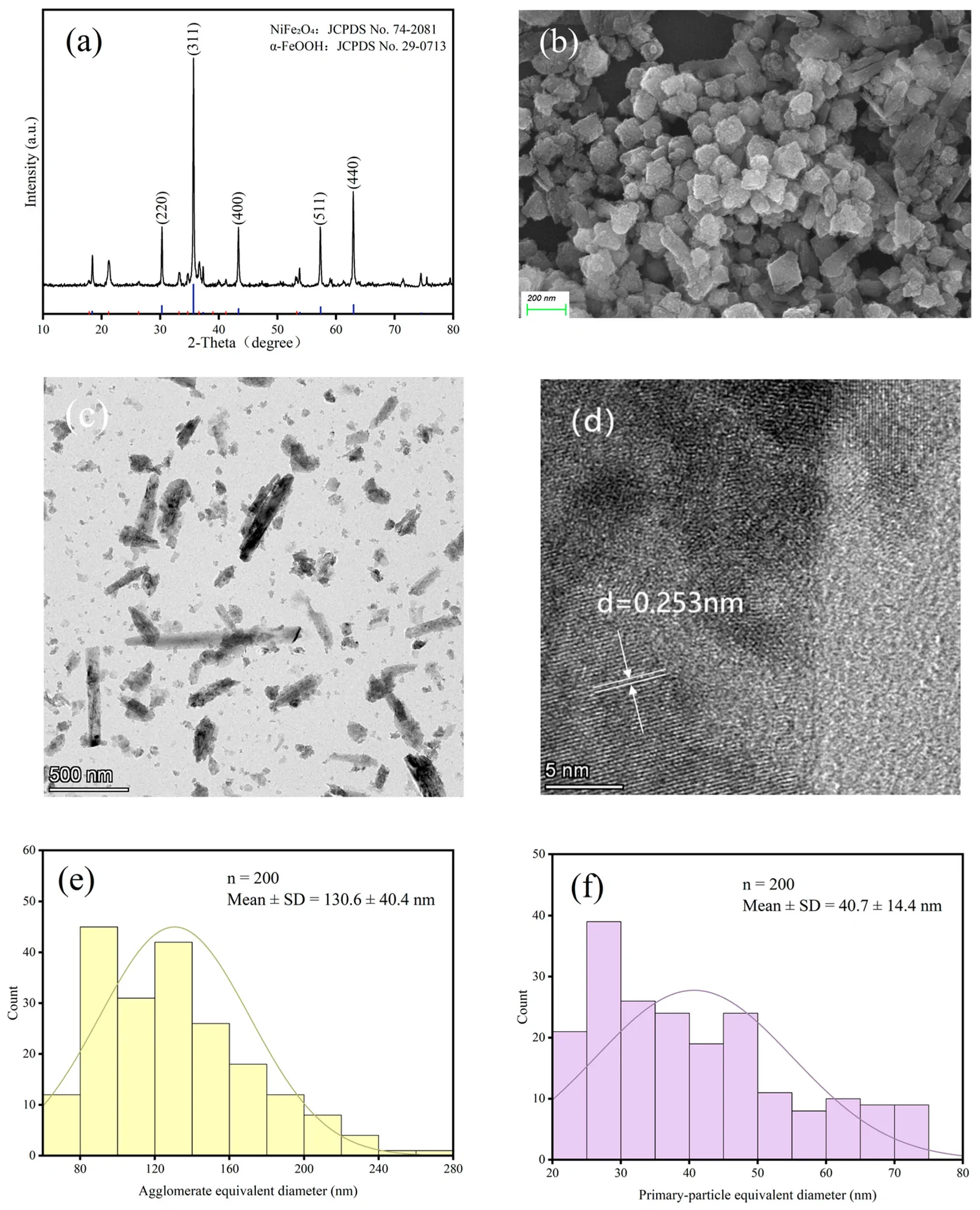
Structural and morphological characterization and quantitative image-based size analysis of the NiFe_2_O_4_ catalyst: (**a**) XRD pattern; (**b**) representative SEM image; (**c**) representative TEM image; (**d**) HRTEM image; (**e**) SEM-derived agglomerate-size distribution; and (**f**) TEM-derived primary-particle-size distribution. The solid curves in panels (**e**,**f**) represent Gaussian fits.

The crystalline structure of the as-synthesized catalyst was characterized by XRD analysis (Figure 1). The XRD data are derived from our previous study [20]. Distinct diffraction peaks appeared at 2θ values of 30.3°, 35.6°, 43.3°, 57.3°, and 63.0°, corresponding to the (220), (311), (400), (511), and (440) crystal planes, respectively (JCPDS No. 74-2081). In addition to the major NiFe_2_O_4_ reflections, weak additional peaks were assigned to a minor α-FeOOH phase, confirming that NiFe_2_O_4_ was the primary crystalline phase, albeit not the sole phase present. The average coherent scattering domain size, calculated from the (311) peak using the Scherrer equation, was approximately 42.4 nm. The SEM image (Figure 1b) reveals relatively large, irregular agglomerated structures rather than isolated primary particles. Quantitative analysis of 200 discernible agglomerates from multiple representative SEM images yielded a mean equivalent diameter of 130.6 ± 40.4 nm (Figure 1e). In contrast, the TEM image (Figure 1c) shows smaller particle-like domains together with elongated features. Quantitative analysis of 200 clearly distinguishable near-equiaxed primary particles from multiple representative TEM images yielded a mean equivalent diameter of 40.7 ± 14.4 nm (Figure 1f). As shown in the high-resolution TEM (HRTEM) image (Figure 1d), the catalyst exhibited well-defined lattice fringes. An interplanar spacing of 0.253 nm was measured, corresponding to the (311) plane of NiFe_2_O_4_.

### 3.2. Evaluation of Different Degradation Systems

**Figure 2:**
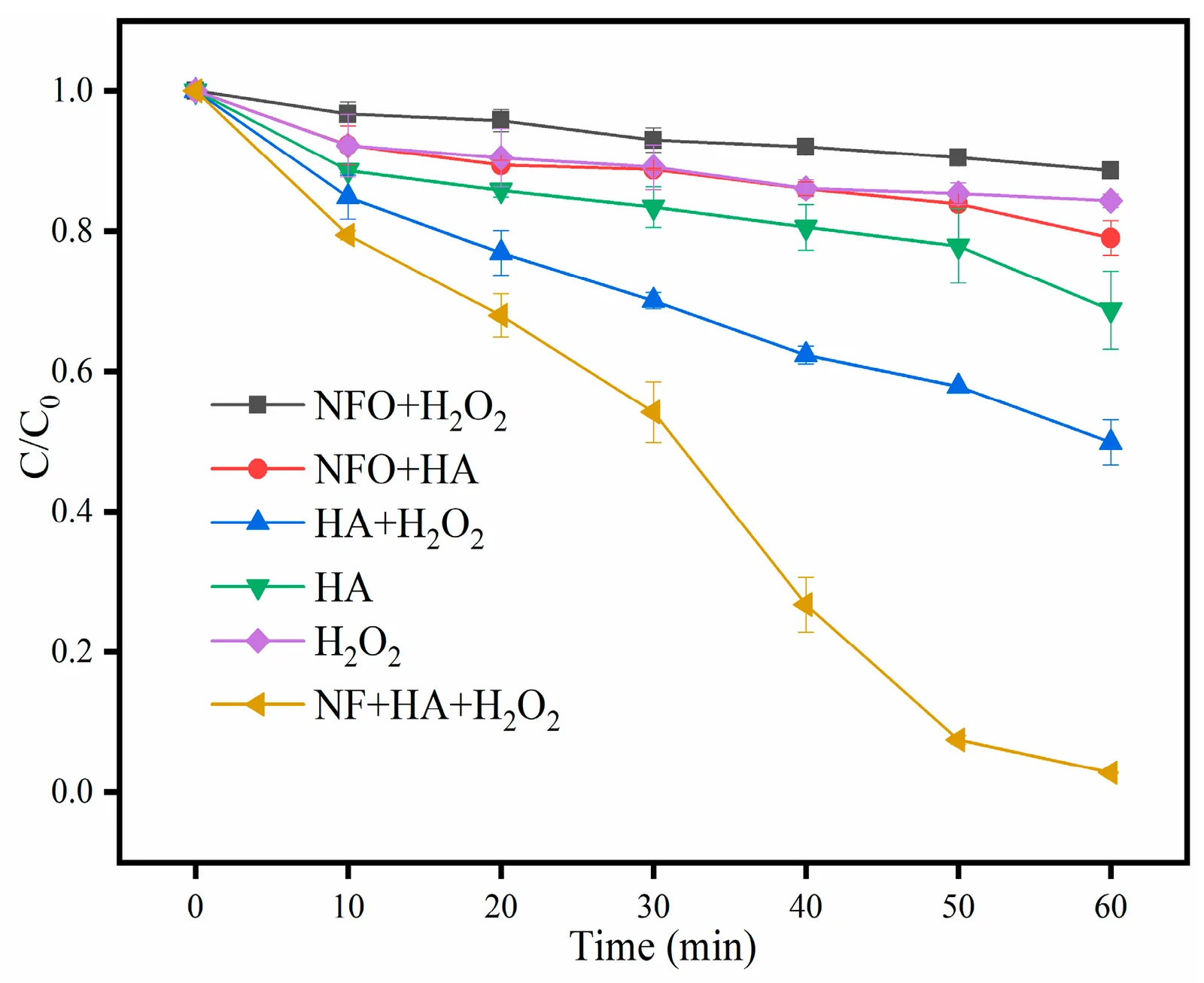
Phenol degradation in different reaction systems. Error bars represent standard deviations (*n* = 3).

To evaluate the catalytic efficiency of the HA-enhanced NiFe_2_O_4_ system, phenol was used as a model pollutant. The baseline experiments were conducted with fixed concentrations of 0.6 g/L catalyst, 50 mg/L phenol, 10 mmol/L H_2_O_2_, and 5 mmol/L HA. Six comparative experiments were conducted, including H_2_O_2_ alone, HA alone, NiFe_2_O_4_/H_2_O_2_, NiFe_2_O_4_/HA, HA/H_2_O_2_, and NiFe_2_O_4_/HA/H_2_O_2_ (Figure 2). In the H_2_O_2_-alone system, phenol degradation reached 17.4%, indicating the limited oxidation ability of H_2_O_2_ alone under the tested conditions [22]. The HA-alone system achieved 35.2% degradation, similarly implying limited removal by HA alone [23]. In the NiFe_2_O_4_/H_2_O_2_ system, phenol degradation also remained marginal under the same conditions. Notably, the NiFe_2_O_4_/HA/H_2_O_2_ system showed the highest performance, achieving 20.8% degradation within the first 10 min and 97.8% degradation after 60 min. These results show that the combined presence of NiFe_2_O_4_, HA, and H_2_O_2_ markedly improved phenol degradation. To further quantify the reaction rates, the degradation data were fitted to a pseudo-first-order kinetic model (−ln(C/C_0_) = kt). The apparent rate constant (k) for the NiFe_2_O_4_/HA/H_2_O_2_ system was calculated to be 0.053 min^−1^ (R^2^ > 0.91), substantially higher than that of the HA-free system (0.003 min^−1^). This approximately 17.6-fold increase in the rate constant further demonstrates the promoting effect of HA under the tested conditions.

### 3.3. Factors Influencing Phenol Degradation in the NiFe_2_O_4_/H_2_O_2_/HA System

#### 3.3.1. Effect of H_2_O_2_ Concentration

**Figure 3:**
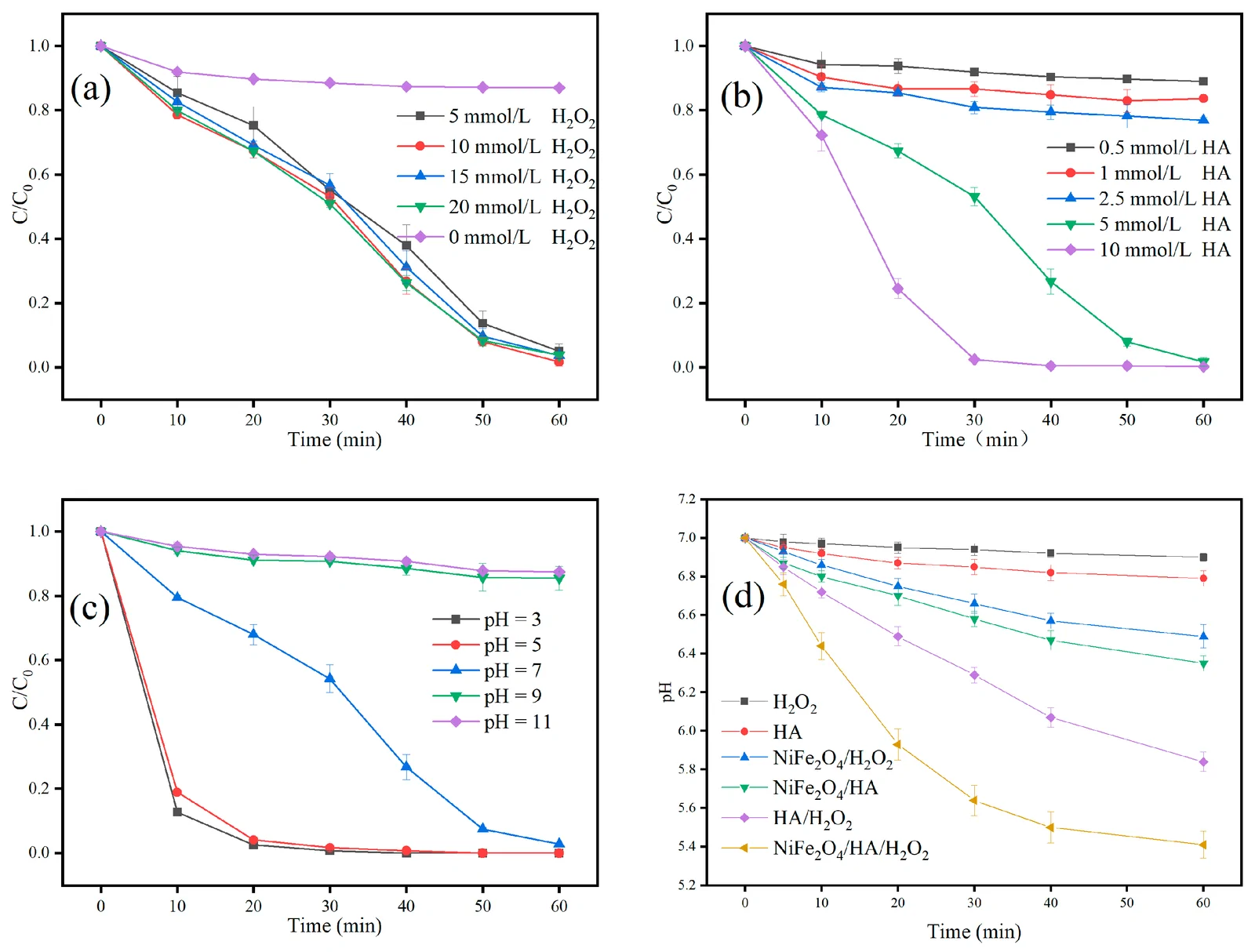
Effects of operational parameters on phenol degradation and temporal pH variation: (**a**) H_2_O_2_ concentration; (**b**) HA concentration; (**c**) initial pH; and (**d**) time-resolved pH profiles of different reaction systems at an initial pH of 7. Error bars represent standard deviations (*n* = 3).

The influence of H_2_O_2_ concentration on phenol degradation within the NiFe_2_O_4_/H_2_O_2_/HA system was investigated (Figure 3a). The experimental parameters were maintained as in Section 3.2, while the H_2_O_2_ concentration was adjusted from 0 to 20 mmol/L. In the absence of H_2_O_2_ (0 mmol/L), the degradation efficiency of phenol was only 13.3%, indicating the indispensable role of H_2_O_2_ in generating active species within this system. Once H_2_O_2_ was introduced, the degradation efficiency increased substantially. However, during the later stage of the reaction (40–60 min), the rate of degradation slowed down, likely because the decreasing H_2_O_2_ concentration resulted in fewer free radicals being generated. At a low H_2_O_2_ (5 mmol/L) concentration, the degradation efficiency of phenol was relatively low. At 10 mmol/L H_2_O_2_, the phenol degradation efficiency reached 97.4% after 60 min. This result indicates that at this concentration, sufficient oxidizing radicals (•OH and O_2_•^−^) are generated, promoting effective degradation. When the H_2_O_2_ concentration was increased to 15 and 20 mmol/L, the degradation efficiencies were 95.7% and 95.9%, respectively. These small endpoint differences relative to the 10 mmol/L system were within the range of experimental variability and were therefore not considered meaningful improvements or declines in degradation performance.

Therefore, 10 mmol/L H_2_O_2_ was selected for subsequent experiments, as it provided comparable phenol degradation at the lowest oxidant dosage among the three concentrations tested.

#### 3.3.2. Effect of HA Concentration

The effect of HA concentration on phenol degradation was subsequently evaluated within the NiFe_2_O_4_/H_2_O_2_/HA system (Figure 3b). Experimental conditions were similar to those in Section 3.2, with HA concentrations varied from 0.5 to 10 mmol/L. When the HA concentration was 0.5 mmol/L, phenol degradation was relatively limited. A slow decline in concentration was observed, with a substantial amount of phenol remaining after 60 min. This indicated that the reductive power provided by a low concentration of HA was insufficient to effectively activate the reaction, resulting in poor degradation performance. At an HA concentration of 1 mmol/L, although the reaction activity was enhanced slightly, the low HA concentration resulted in insufficient generation of reactive species, leading to a low degradation efficiency of 52.6%. When the HA concentration was increased to 2.5 mmol/L, the phenol degradation efficiency improved to 68.3%. At an HA concentration of 5 mmol/L, phenol degradation reached 97.8% within 60 min. This HA concentration corresponds to approximately 165 mg/L NH_2_OH, equivalent to about 347 mg/L NH_2_OH·HCl as dosed. Increasing the HA concentration to 10 mmol/L further accelerated phenol degradation, achieving 99.8% removal within 30 min. However, the higher HA dosage may increase residual HA and nitrogen-containing transformation products. Therefore, 5 mmol/L HA was selected as a compromise between degradation performance and reductant consumption.

#### 3.3.3. Effect of Initial pH

Conventional Fenton reactions typically occur under acidic conditions. During phenol degradation, pH significantly affects the speciation of intermediate products. Accordingly, the system’s degradation performance was examined across an initial solution pH range of 3.0 to 11.0. As depicted in Figure 3c, the initial solution pH was found to have a significant influence on the NiFe_2_O_4_/H_2_O_2_/HA process. The initial pH of the solution was adjusted within the 3.0–11.0 range using 0.1 mol/L H_2_SO_4_ or 0.1 mol/L NaOH, and all pH values were monitored with a Raycom (PHS-3C) pH meter.

The system was highly effective in acidic environments (Figure 3c). Specifically, at an initial pH of 3.0, degradation efficiency reached 87.2% within 10 min, with complete removal observed by 30 min. As the pH increased to 5.0, a slight reduction in the initial rate was noted (81.4% at 10 min), with near-complete degradation occurring within 40 min. This observation can be attributed to the acidic environment, which facilitates the formation of Fe^2+^, a species that plays a crucial role in catalyzing H_2_O_2_ decomposition [24]. At an initial pH of 7, the phenol degradation efficiency reached 97.8% after 60 min. However, under alkaline conditions (pH 9 to 11), the degradation efficiency declined significantly. This reduction could be due to the precipitation of Fe^3+^ as iron hydroxide in highly alkaline environments, as well as the quenching of free radicals, both of which inhibit the Fenton reaction. Additionally, these hydroxide complexes tend to deposit on the catalyst surface, hindering the release of Fe^2+^ into the solution [25]. This process inevitably weakens the activation of H_2_O_2_ by Fe^2+^. Consistent with previous studies, the formation of iron hydroxide precipitates under strongly alkaline conditions may be unfavorable for the Fenton-like reaction [26].

The time-resolved pH profiles of the different reaction systems are shown in Figure 3d. The pH of the H_2_O_2_-only and HA-only systems decreased slightly from 7.00 to 6.90 and 6.79, respectively, after 60 min. In comparison, the pH of the HA/H_2_O_2_ system decreased to 5.84, whereas that of the complete NiFe_2_O_4_/H_2_O_2_/HA system decreased to 5.41. These results indicate that although the initial pH was set to 7, the reaction solutions became acidic over time, especially in the presence of both HA and H_2_O_2_.

### 3.4. Metal Leaching and Homogeneous–Heterogeneous Contributions

To evaluate whether the system behaves as a heterogeneous surface-catalyzed reaction or involves homogeneous processes, the leaching of dissolved Fe during the reaction was quantified. The dissolved Fe concentration increased progressively from 0.2 mg/L at 10 min to 1.3 mg/L at 30 min and 4.1 mg/L at 60 min. For comparison, the dissolved Fe concentration reached only approximately 0.5 mg/L after 60 min in the absence of HA, indicating that HA promoted Fe dissolution from the catalyst.

**Figure 4:**
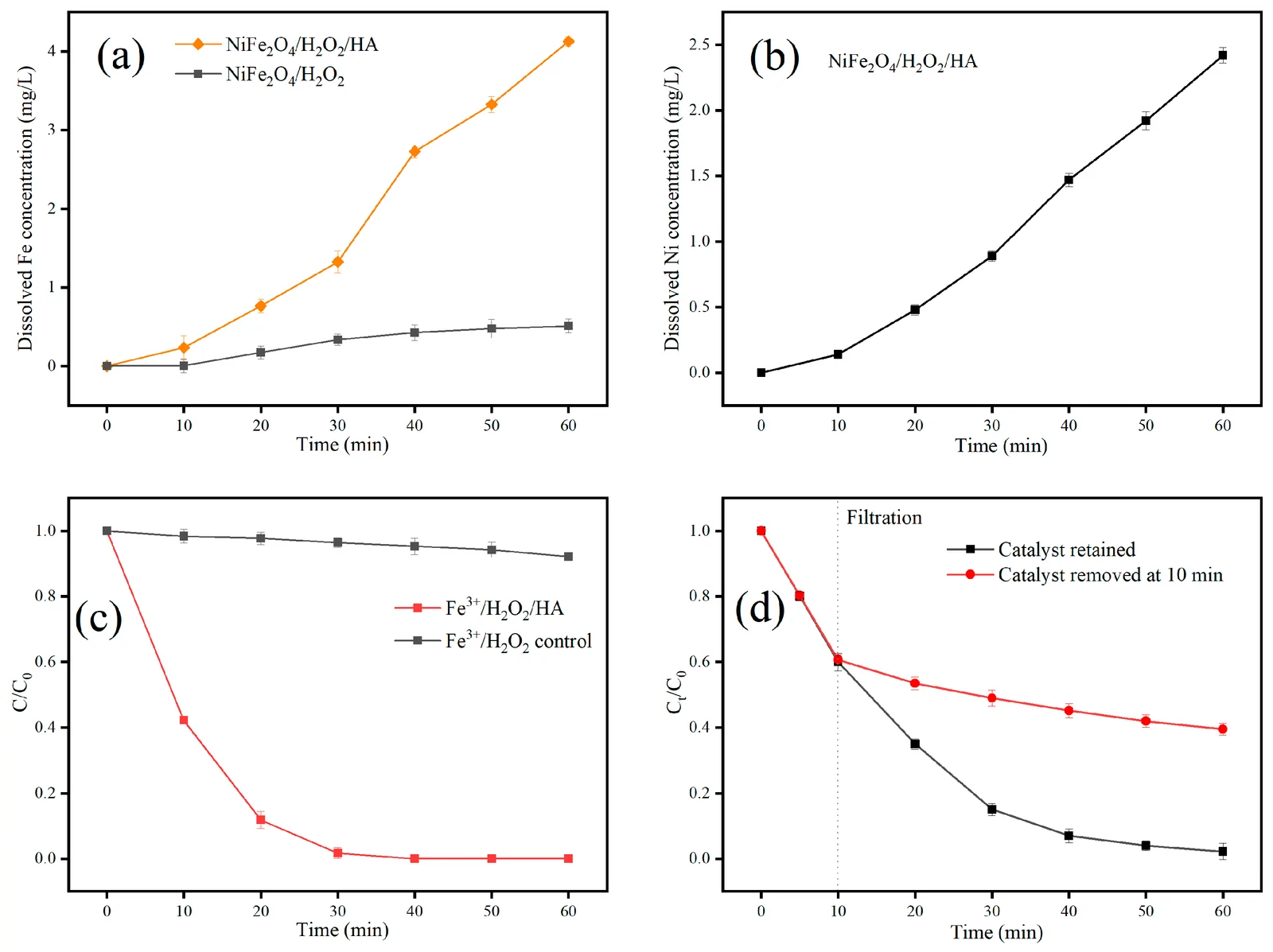
Metal leaching and reaction-phase contributions in the NiFe_2_O_4_/H_2_O_2_/HA system: (**a**) dissolved Fe concentrations with and without HA; (**b**) dissolved Ni concentration over time; (**c**) phenol degradation in the Fe^3+^/H_2_O_2_/HA and Fe^3+^/H_2_O_2_ control systems; and (**d**) catalyst-removal experiment conducted at 10 min. Error bars represent standard deviations (*n* = 3).

To assess the contribution of dissolved Fe species, a Fe^3+^/H_2_O_2_/HA control system was tested using 50 mL of 50 mg/L phenol solution, 4.1 mg/L Fe^3+^, 10 mmol/L H_2_O_2_, and 10 mmol/L HA. As shown in Figure 4c, the Fe^3+^/H_2_O_2_/HA control system exhibited rapid phenol degradation. Specifically, phenol degradation reached 58.1% within 10 min and 97.1% after 30 min. In contrast, the Fe^3+^/H_2_O_2_ control system achieved only 7.9% phenol degradation after 60 min, indicating limited activity in the absence of HA. These results indicate that dissolved Fe species can contribute to phenol degradation in the presence of HA. However, this control experiment alone does not quantify their contribution in the complete catalyst-containing system. Dissolved Ni was also detected in the complete reaction system, with its concentration gradually increasing to 2.42 ± 0.06 mg/L after 60 min. The simultaneous release of Fe and Ni suggests that metal leaching should be considered when evaluating catalyst stability and practical applicability.

To further distinguish the contributions of dissolved species and the solid catalyst, the NiFe_2_O_4_ catalyst was removed after 10 min. Phenol degradation continued after catalyst removal, indicating that dissolved species contributed to the reaction. However, the catalyst-retained system showed higher continued degradation, indicating that the heterogeneous catalyst also remained involved. Therefore, both homogeneous and heterogeneous processes contributed to phenol degradation.

Nevertheless, the observed Fe and Ni leaching poses a practical limitation, and improved metal immobilization or appropriate post-treatment would be required before practical application.

### 3.5. XPS Analysis of NiFe_2_O_4_ Before and After Reaction

**Figure 5:**
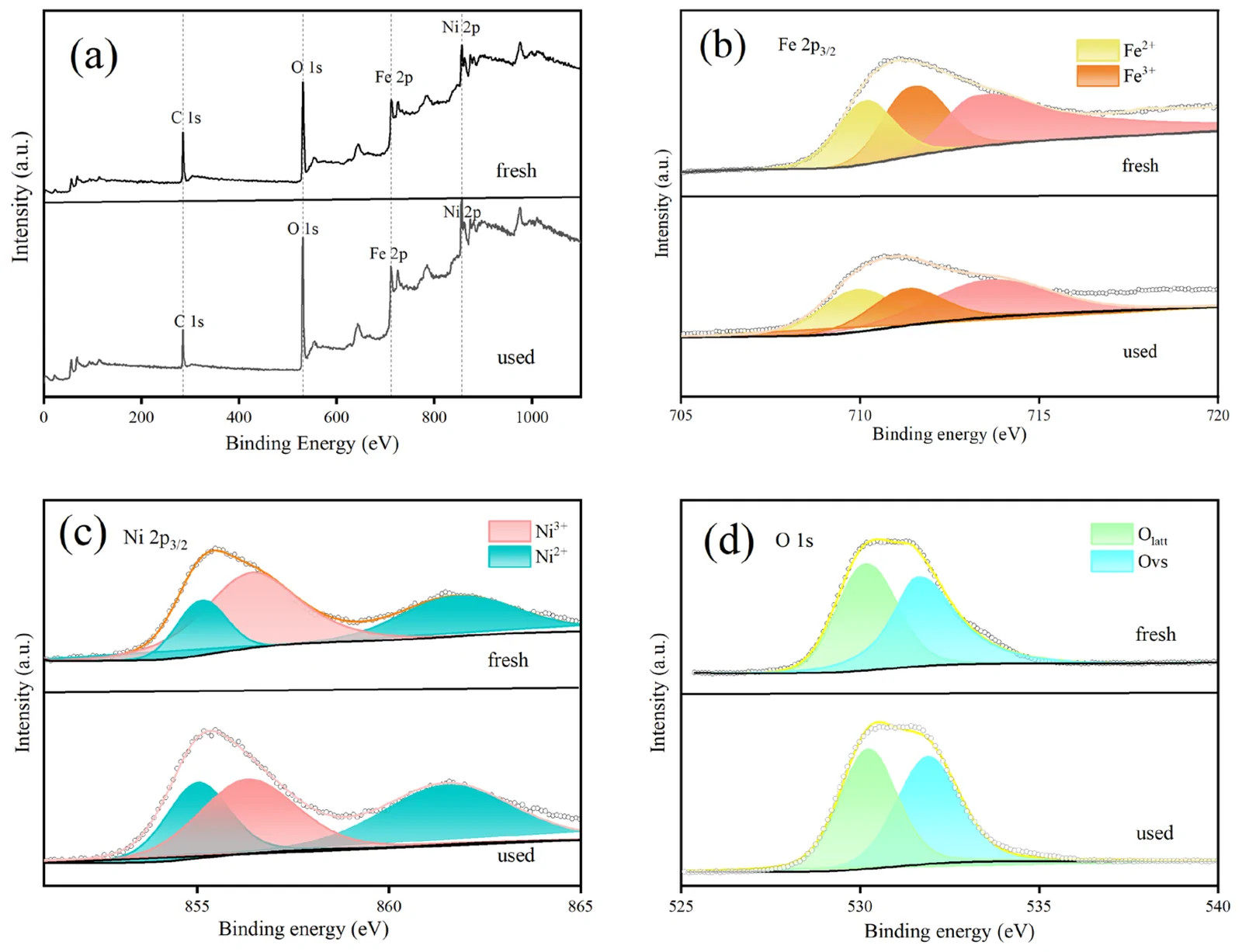
XPS spectra of fresh and used NiFe_2_O_4_: (**a**) survey spectra; (**b**) Fe 2p; (**c**) Ni 2p; and (**d**) O 1s spectra.

XPS was employed to elucidate the role of the reducing agent, providing a detailed analysis of the catalyst’s surface elemental composition before and after the reaction (Figure 5). As shown in Figure 5a, the surface of the NiFe_2_O_4_ primarily contained nickel, iron, oxygen, and exogenous carbon, with no significant changes in elemental distribution. The XPS data of fresh NiFe_2_O_4_ were derived from our previous study [20]. The Fe 2p XPS spectra (Figure 5b) reveal two main characteristic peaks at 710.1 eV and 711.4 eV, corresponding to Fe^2+^ and Fe^3+^ [27,28], respectively. Before the reaction, the relative proportions of Fe^2+^ and Fe^3+^ were 49.1% and 50.9%, respectively. After the reaction, the proportion of Fe^2+^ increased to 53.6%, while Fe^3+^ decreased to 46.4%. These changes are consistent with the formation or regeneration of reduced Fe species in the presence of HA.

The Ni 2p spectra of NiFe_2_O_4_ (Figure 5c) revealed three distinct peaks of Ni on the catalyst surface. Peaks at 855.1 eV and 861.8 eV correspond to Ni^2+^ [29], while the peak at 856.4 eV corresponds to Ni^3+^ species [30]. Following the reaction, the proportion of Ni^2+^ increased from 52.1% to 63.4%, accompanied by a decrease in Ni^3+^ from 47.9% to 36.6%. The increase in the Ni^2+^ proportion is consistent with the formation or regeneration of reduced Ni species during the reaction. This change may result from direct reduction by HA, Fe^2+^-mediated reduction of Ni^3+^, and/or electron redistribution through Fe–O–Ni linkages; however, the present ex situ XPS data cannot distinguish among these possible pathways. Overall, the observed changes in the Fe and Ni oxidation states indicate surface metal-valence transformations during the reaction, but do not establish the specific rates or direct pathways of the corresponding redox cycles.

As shown in Figure 5d, the O 1s spectra of NiFe_2_O_4_ before and after the reaction display two distinct oxygen species. The spectra were deconvoluted into two main peaks: surface lattice oxygen (Olatt) at 530.1 eV and oxygen vacancies (O_Vs_) at 531.6 eV. Previous studies have reported the involvement of surface lattice oxygen in metal-oxide redox reactions [31] and oxygen-vacancy-associated sites in surface Fenton chemistry [32]. However, in this study, the relative proportions of O_latt_ and OVs changed only slightly, from 50.5% to 50.1% and from 49.5% to 49.9%, respectively. These marginal changes suggest that HA-induced surface reconstruction or enhanced adsorption of reactive species was not substantial under the tested conditions.

### 3.6. Detection of Active Radicals

Radical scavenging experiments were conducted to determine the types and roles of reactive oxygen species [6] responsible for phenol degradation in the NiFe_2_O_4_/H_2_O_2_/HA system. Specific scavengers were employed, including 2 mmol/L tert-butyl alcohol (TBA) to quench •OH radicals and 2 mmol/L benzoquinone (BQ) to inhibit O_2_•^−^ radicals. Additionally, a blank control group (without a scavenger) was included to evaluate the influence of radicals on the reaction. The results indicated that TBA markedly inhibited the degradation of phenol, with only 34.8% of phenol degraded after 60 min. Upon addition of BQ, the degradation efficiency of phenol dropped dramatically to 28.4% after 60 min. These results suggest that both •OH and O_2_•^−^ contributed substantially to phenol degradation under the tested conditions.

**Figure 6:**
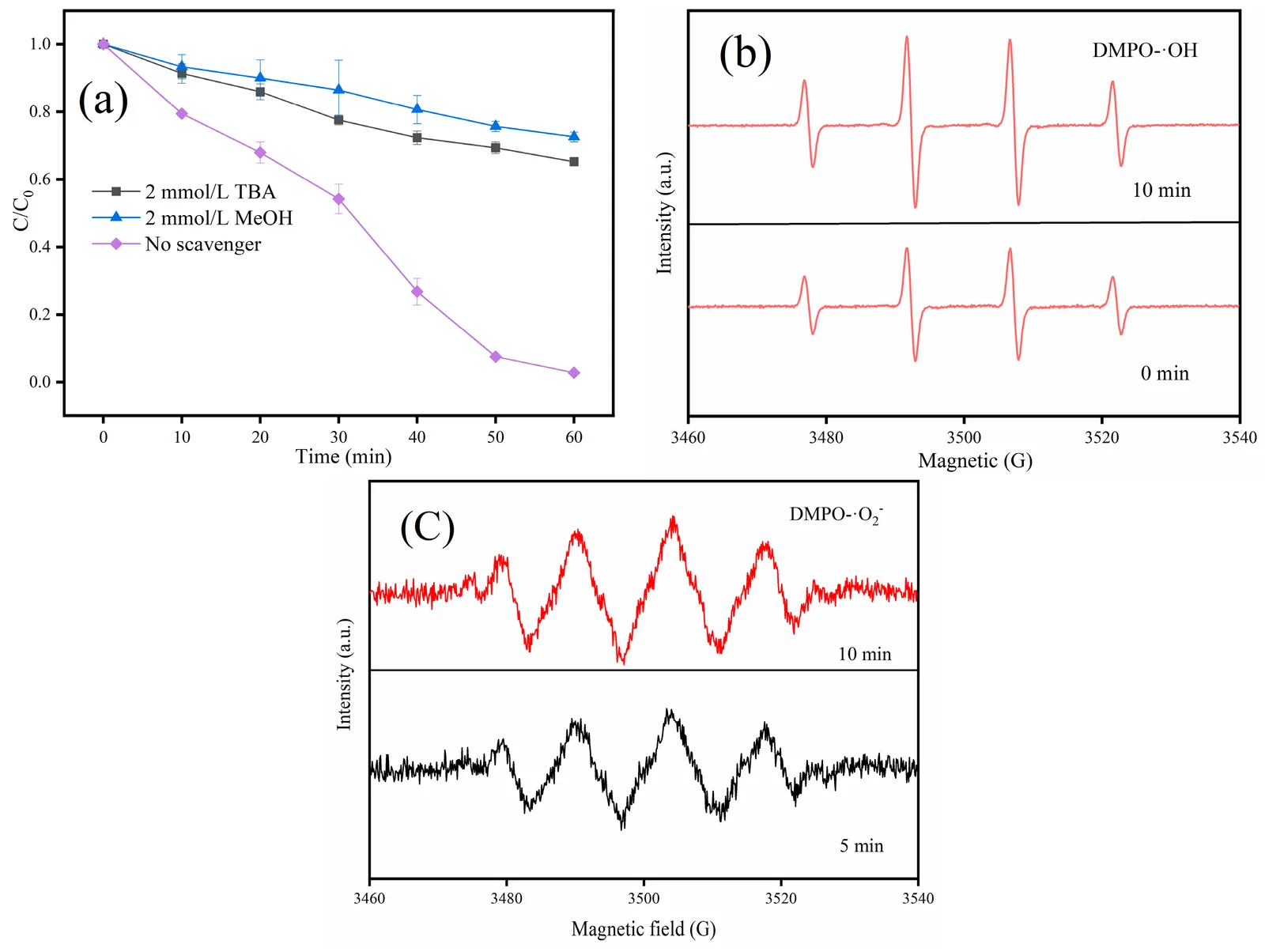
Reactive-species identification in the NiFe_2_O_4_/H_2_O_2_/HA system: (**a**) effects of TBA and BQ on phenol degradation; (**b**) DMPO–•OH EPR spectra recorded at 0 and 10 min; and (**c**) DMPO–O_2_•^−^ EPR spectra recorded at 5 and 10 min. In panel (**a**), error bars represent standard deviations (*n* = 3).

Additionally, electron paramagnetic resonance (EPR) spectroscopy was employed to detect and analyze the generation of ROS during the catalytic reaction. For the detection of •OH and O_2_•^−^ radicals, 5,5-dimethyl-1-pyrroline N-oxide (DMPO) was used as a spin trapping reagent. As shown in Figure 6b,c, DMPO–•OH signals were observed at 0 and 10 min, whereas DMPO–O_2_•^−^ signals were detected at 5 and 10 min. The observed signals confirm the generation of •OH and O_2_•^−^ in the NiFe_2_O_4_/H_2_O_2_/HA system. Taken together, the scavenging and EPR results indicate that •OH and O_2_•^−^ were the predominant reactive species under the tested conditions.

### 3.7. Proposed Reaction Mechanism

**Figure 7:**
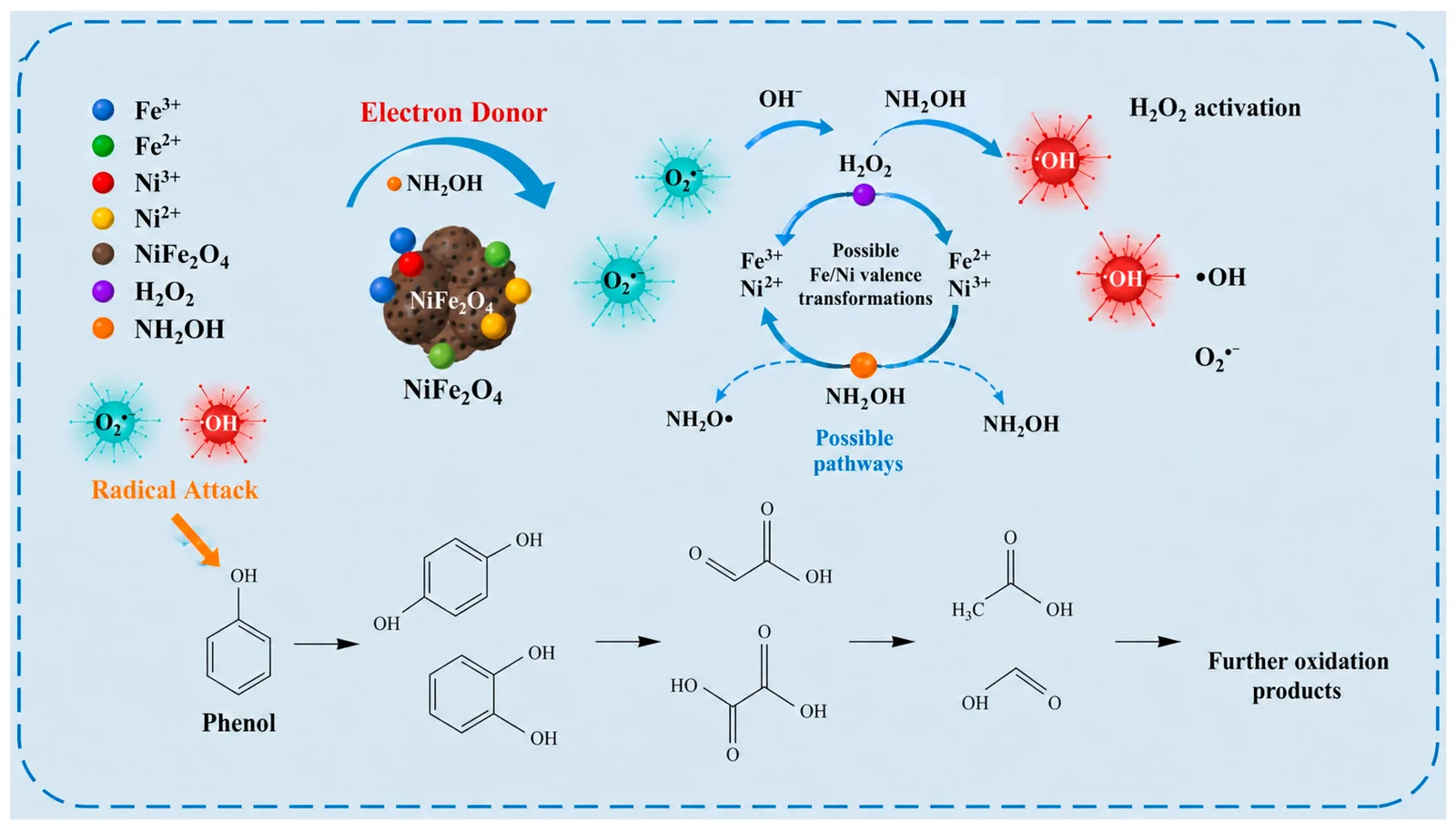
Proposed reaction mechanism for phenol degradation in the NiFe_2_O_4_/H_2_O_2_/HA system.

Based on the scavenging and EPR results, •OH and O_2_•^−^ were involved in phenol degradation, and a possible reaction mechanism is proposed below (see Figure 7). As the reaction pH decreased, both surface metal transformations and the release of dissolved metal species could contribute to H_2_O_2_ activation. HA can promote the formation or regeneration of Fe^2+^ species, thereby facilitating H_2_O_2_ activation. Fe^3+^ and Fe^2+^ species may participate in H_2_O_2_ activation, generating reactive species that subsequently oxidize phenol.

The XPS results are consistent with the formation or regeneration of reduced Fe and Ni species during the reaction. For Ni species, the observed increase in Ni^2+^ may arise from direct reduction by HA, Fe^2+^-mediated reduction of Ni^3+^, and/or electron redistribution through Fe–O–Ni linkages; however, the present results cannot distinguish among these possible pathways. Furthermore, NH_2_OH has been reported to react with H_2_O_2_ through a single-electron-transfer pathway to generate •OH [33].

Based on these findings, the possible reaction pathways are summarized in Equations (1)–(11) below.

Fe^2+^/Ni^2+^ + H_2_O_2_ → Fe^3+^/Ni^3+^ + •OH + OH^−^ (1)

Fe^3+^/Ni^3+^ + H_2_O_2_ → Fe^2+^/Ni^2+^ + HO_2_• + H^+^ (2)

Fe^3+^ + HO_2_• → Fe^2+^ + O_2_ + H^+^ (3)

Fe^2+^ + HO_2_• + H^+^ → Fe^3+^ + H_2_O_2_ (4)

H_2_O_2_ + Fe^3+^ + 2OH^−^ → Fe^2+^ + O_2_•^−^ + 2H_2_O (5)

Fe^3+^/Ni^3+^ + O_2_•^−^ → Fe^2+^/Ni^2+^ + O_2_ (6)

Fe^2+^ + O_2_•^−^ + 2H^+^ → Fe^3+^ + H_2_O_2_ (7)

H_2_O_2_ + NH_3_OH^+^ → •OH + NH_3_O•^+^ + H_2_O (8)

H_2_O_2_ + NH_3_O•^+^ → •OH + HNO + H^+^ + H_2_O (9)

Fe^3+^ + NH_2_OH ⇆ NH_2_O• + Fe^2+^ + H^+^ (10)

Fe^3+^ + NH_2_O• → Fe^2+^ + HNO + H^+^ (11)

### 3.8. Transformation of HA-Derived Nitrogen Species

**Figure 8:**
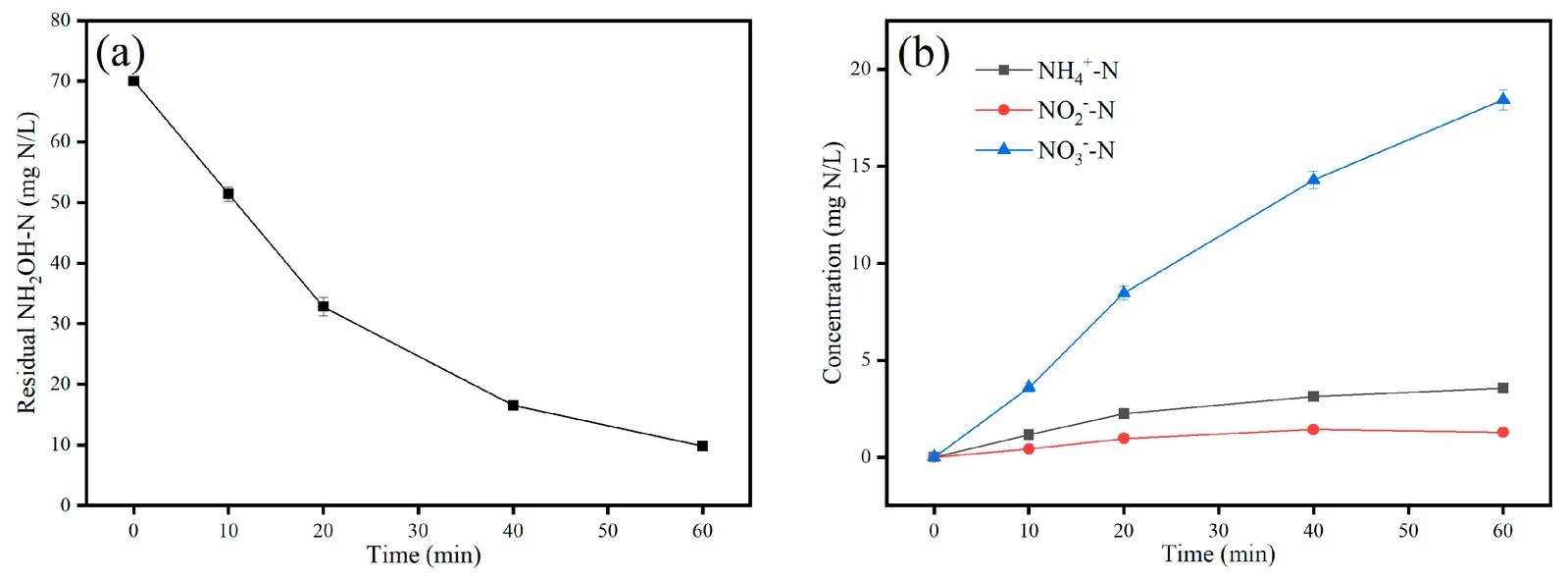
Temporal variations in (**a**) residual NH_2_OH-N and (**b**) quantified nitrogen-containing products in the NiFe_2_O_4_/H_2_O_2_/HA system. Error bars represent standard deviations (*n* = 3).

To evaluate the transformation of HA, residual NH_2_OH-N and quantified nitrogen-containing products were monitored during the reaction (Figure 8). The NH_2_OH-N concentration decreased from 70.0 ± 0.2 to 9.8 ± 0.4 mg N/L within 60 min, corresponding to 86.0% consumption. Meanwhile, NO_3_^−^-N was the predominant quantified aqueous nitrogen product, reaching 18.42 ± 0.52 mg N/L, whereas NH_4_^+^-N and NO_2_^−^-N reached 3.55 ± 0.18 and 1.28 ± 0.09 mg N/L, respectively.

At 60 min, the sum of the quantified dissolved nitrogen species was 33.05 ± 0.70 mg N/L, corresponding to 47.2% of the initial NH_2_OH-N. Therefore, these measurements do not constitute a complete nitrogen balance, and the formation of unmeasured nitrogen species should not be inferred without direct analytical evidence. The residual HA and nitrogen-containing products should be considered when evaluating reagent consumption and environmental applicability.

### 3.9. Intermediate Product Analysis

**Figure 9:**
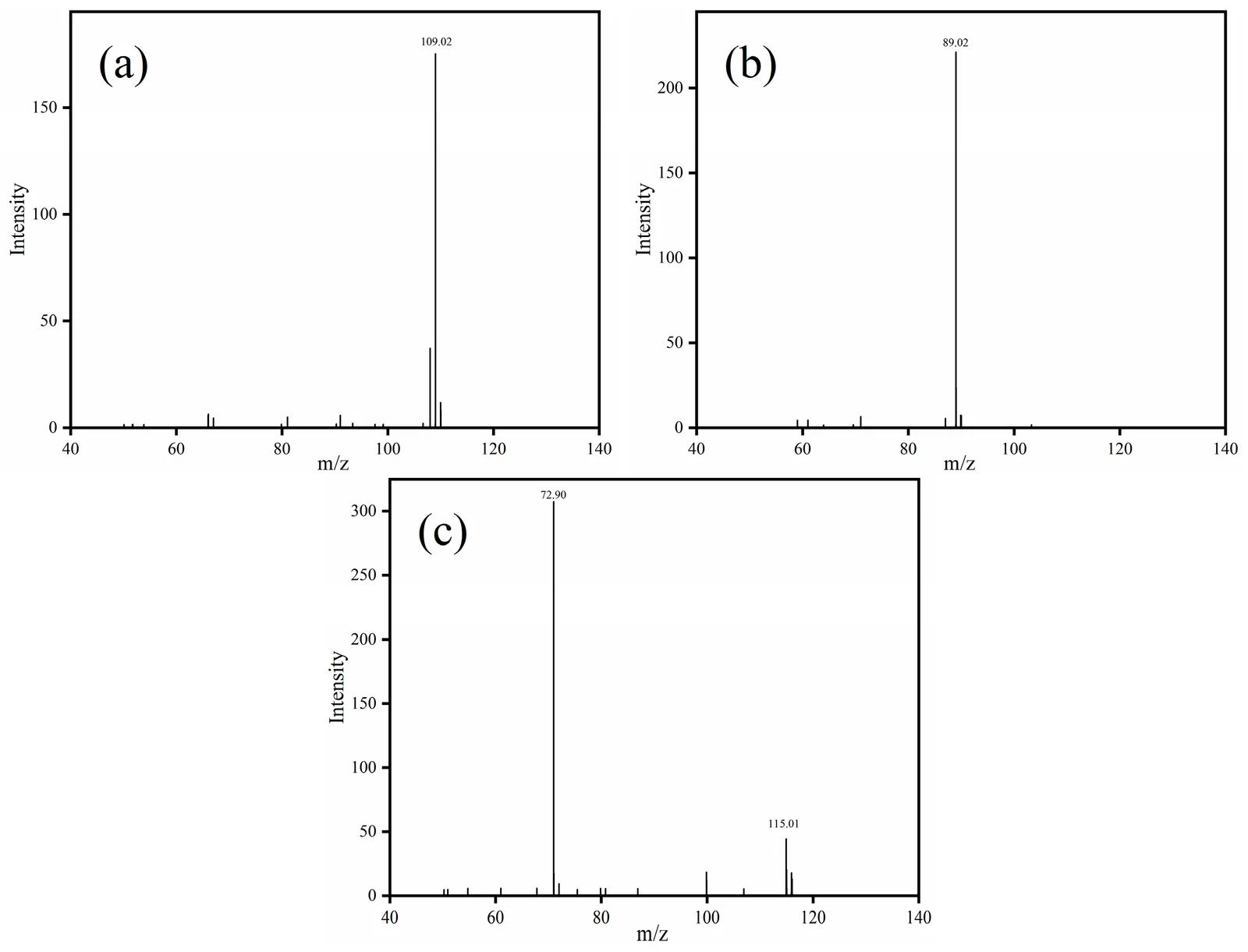
LC–MS spectra of the proposed phenol-degradation intermediates: (**a**) *m*/*z* 109.02; (**b**) *m*/*z* 89.02; and (**c**) *m*/*z* 72.90.

The intermediate products formed during phenol degradation in the NiFe_2_O_4_/H_2_O_2_/HA system were analyzed using LC-MS. Samples were collected after 30 min of reaction for analysis. Based on the mass-to-charge ratios (*m*/*z*), the intermediate products were tentatively assigned. As shown in Figure 9, several types of intermediate products were detected. In particular, the signal at *m*/*z* 109.02 was tentatively assigned to dihydroxybenzene isomers, including para-benzenediol and ortho-benzenediol (C_6_H_6_O_2_). Additionally, the signals at *m*/*z* 89.02 and 72.90 were tentatively assigned to oxalic acid (H_2_C_2_O_4_) and glyoxylic acid (C_2_H_2_O_3_), respectively.

**Figure 10:**
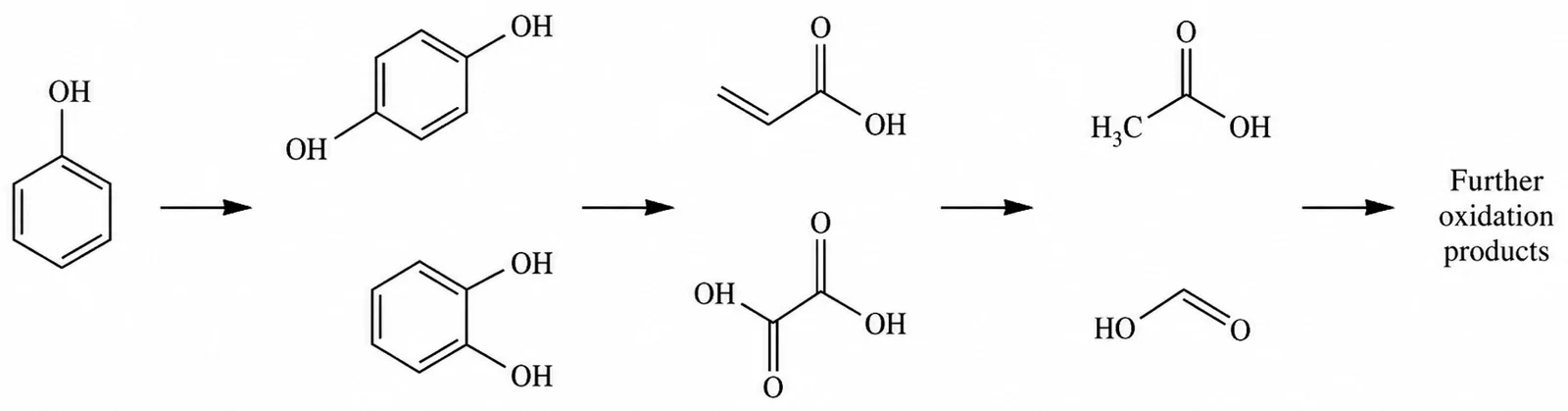
Proposed transformation pathway of phenol in the NiFe_2_O_4_/H_2_O_2_/HA system.

As illustrated in Figure 10, a possible degradation pathway for phenol degradation is proposed, which appears to proceed via two primary routes. In the early stages of the reaction, phenol undergoes oxidation to form para- and ortho-benzenediol. These intermediates are subsequently oxidized into low-molecular-weight organic acids, such as acetoacetic acid and oxalic acid. These products are further decomposed into smaller molecules such as acetic acid and formic acid. These low-molecular-weight products may undergo further oxidation; however, complete mineralization was not evaluated because TOC was not measured in this study.

### 3.10. Catalyst Stability and Reusability

**Figure 11:**
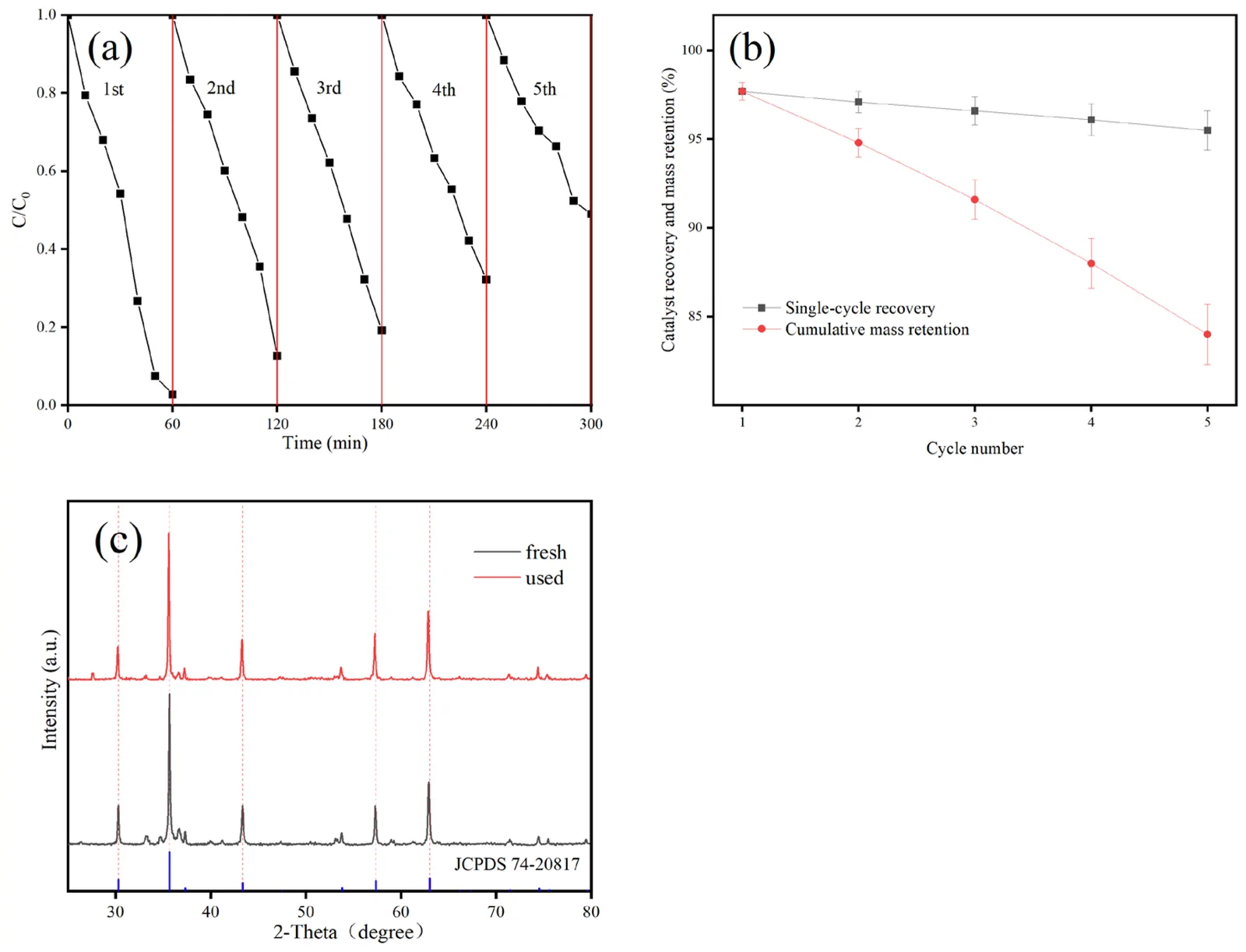
Catalyst reusability and recovery: (**a**) phenol degradation during consecutive reaction cycles; (**b**) single-cycle catalyst recovery yield and cumulative mass retention; and (**c**) XRD patterns of the fresh and used catalysts. In panels (**a**,**b**), error bars represent standard deviations (*n* = 3).

The reusability of the NiFe_2_O_4_ catalyst was evaluated through consecutive reaction cycles, with the catalyst recovered by centrifugation after each cycle. The phenol-degradation performance, catalyst recovery, and structural changes during reuse are shown in Figure 11. The catalyst’s reusability was evaluated, showing a gradual decline in phenol degradation efficiency over successive cycles (Figure 11a). The efficiency decreased from 87.4% in the first cycle to 80.8%, 68.8%, and 52.1% in the second, third, and fourth cycles, respectively. The decrease in activity may be associated with catalyst mass loss, metal leaching, and possible blockage of active sites by adsorbed reaction products.

The single-cycle catalyst recovery yield remained between 95.5% and 97.7%, while the cumulative catalyst mass retention decreased to 84.0 ± 1.7% after five cycles, indicating that repeated handling caused a gradual but measurable loss of catalyst mass. Additionally, the XRD pattern of the used catalyst was analyzed to evaluate its structural stability (Figure 11c). The major crystalline structure was largely retained after reuse, with only a slight decrease in diffraction intensities and no obvious new crystalline phases observed. These XRD results suggest that the decline in catalytic performance was not accompanied by a major crystalline-phase transformation. Nevertheless, the observed activity loss, catalyst mass loss, and metal leaching remain important limitations for repeated use.

## 4. Conclusions

This study developed an HA-enhanced NiFe_2_O_4_/H_2_O_2_ Fenton-like system that achieved 97.8% phenol degradation within 60 min at an initial pH of 7. XPS analysis revealed increased proportions of Fe^2+^ and Ni^2+^ after the reaction, consistent with the formation or regeneration of reduced metal species. However, these ex situ changes do not directly confirm accelerated Fe^3+^/Fe^2+^ or Ni^3+^/Ni^2+^ redox cycling. Scavenging and EPR experiments identified •OH and O_2_•^−^ as the predominant reactive species, while metal leaching, dissolved-Fe control, and catalyst-removal tests indicated contributions from both heterogeneous and homogeneous pathways.

Several practical limitations should be noted, including Fe and Ni leaching, residual HA-derived nitrogen species, catalyst mass loss, and the progressive performance decline upon reuse. Future work should focus on improving metal immobilization and catalyst stability, minimizing HA consumption and nitrogen-containing byproducts.

## Data Availability

The raw data supporting the conclusions of this article will be made available by the authors on request.
